# Hands-On Experiences in Deploying Cost-Effective Ambient-Assisted Living Systems

**DOI:** 10.3390/s150614487

**Published:** 2015-06-18

**Authors:** Athanasios Dasios, Damianos Gavalas, Grammati Pantziou, Charalampos Konstantopoulos

**Affiliations:** 1 School of Science and Technology, Hellenic Open University, Patras GR-26335, Greece; E-Mail: dgavalas@aegean.gr; 2 Department of Cultural Technology and Communication, University of the Aegean, Mytilene GR-81100, Greece; 3 Department of Informatics, Technological Educational Institution of Athens, Athens GR-12243, Greece; E-Mail: pantziou@teiath.gr; 4 Department of Informatics, University of Piraeus, Piraeus GR-18534, Greece; E-Mail: konstant@unipi.gr

**Keywords:** ambient assisted living, smart home, elderly, monitoring, wireless sensor network, Arduino, ZigBee, XBee

## Abstract

Older adults’ preferences to remain independent in their own homes along with the high costs of nursing home care have motivated the development of Ambient Assisted Living (AAL) technologies which aim at improving the safety, health conditions and wellness of the elderly. This paper reports hands-on experiences in designing, implementing and operating UbiCare, an AAL based prototype system for elderly home care monitoring. The monitoring is based on the recording of environmental parameters like temperature and light intensity as well as micro-level incidents which allows one to infer daily activities like moving, sitting, sleeping, usage of electrical appliances and plumbing components. The prototype is built upon inexpensive, off-the-shelf hardware (e.g., various sensors, Arduino microcontrollers, ZigBee-compatible wireless communication modules) and license-free software, thereby ensuring low system deployment costs. The network comprises nodes placed in a house’s main rooms or mounted on furniture, one wearable node, one actuator node and a centralized processing element (coordinator). Upon detecting significant deviations from the ordinary activity patterns of individuals and/or sudden falls, the system issues automated alarms which may be forwarded to authorized caregivers via a variety of communication channels. Furthermore, measured environmental parameters and activity incidents may be monitored through standard web interfaces.

## 1. Introduction

Recent advances in medical sciences along with the declining birth rate in the developed world is projected to exacerbate the phenomenon of population aging in the coming years. This effect is expected to challenge the viability of health and welfare systems, requiring substantial public and private financial funding for the maintenance of institutions and infrastructure such as health and elderly care centers or nursing homes. It is, therefore, urgent to investigate solutions that will prolong the independent living of elderly people at home, deferring their moving to care centers as long as possible. These solutions should take into account factors that compromise the level of safe living of senior citizens. Such factors include sudden ailments and falls, which commonly cause injuries or loss of consciousness, making it impossible for elderly who live alone to call for help.

In recent years, researchers have developed a variety of assistive technologies based on the emerging “ambient intelligence” paradigm. Ambient intelligence aims at empowering human capabilities by the means of digital environments that are sensitive, adaptive, and responsive to human needs [1,2]. This vision of daily environments enables innovative human–machine interactions characterized by pervasive, unobtrusive and anticipatory communications. Assisted living technologies based on ambient intelligence support the development of the so-called ambient-assisted living (AAL) systems. AAL can effectively improve the safety, health conditions and wellness of elderly individuals. These goals are supported by Wireless Sensor Network (WSN) infrastructures aiming at the continuous monitoring of elderly status, early diagnosis of potential health deterioration and detection of hazardous events. Among others, AAL technologies have been utilized in: mobile emergency response systems [3]; fall detection systems [4,5,6]; video surveillance systems [7,8]; activities of daily living (ADL) monitoring systems [9]; reminders issuing systems (e.g., for medication intake) [10]; chronic disease management and rehabilitation [11]; mobility and automation assistive tools [12]; systems that ease the connection and communication with peers, family and friends [13].

However, most existing AAL systems are based either on intrusive and costly equipment (e.g., cameras, wearables) or on sensor nodes which are relatively complex to configure, program and extend (e.g., TinyOS platforms). Recent developments in embedded systems and microcontroller technologies have opened up new opportunities for the automation industry. First evidence for the suitability of microcontroller platforms in smart home automation applications already exists [14,15]; nevertheless, the potential of microcontrollers for building robust and cost-effective AAL systems remains open to investigation. Hands-on experiences are missing in extending the capabilities of microcontrollers with appropriate low-cost off-the-shelf communication, sensory and actuation components so as to effectively support AAL services. Furthermore, energy management issues need to be tackled to improve systems’ endurance, while privacy concerns should be convincingly addressed to increase user acceptance.

Herein, we present UbiCare, an AAL system based on a cost-effective WSN installation. The key objective of UbiCare is to support the safe independent living of elderly living in their home environment; also to mitigate the stress caused to elderly individuals living in non-supervised areas. The main function of the proposed system is to record the daily activity of the elderly (e.g., presence/movement in specific home areas, sleeping, seating, usage of electrical appliances or sanitary facilities) and environmental monitoring (temperature, humidity, light intensity). Prospectively, significant deviations from the “normal” activity pattern (for instance, prolonged immobility on the bed or detection of prolonged presence at home without food consumption) could be interpreted as evidence of incapacitation or reason to issue alarm. UbiCare may also issue alarms to authorized caregivers (e.g., relatives and/or doctors) in the event of fall detection, thereby improving the achievable level of security and ensuring immediate nursing treatment of such incidents; alarms may be implemented by a variety of methods, e.g., automated sending of SMS, emails or voice calls. Activity information is recorded in a web database and visualized in an intelligible form via a web interface.

The main contribution of this paper lies in the documentation of hands-on experiences in designing, implementing and operating AAL systems through utilizing inexpensive equipment (effectively, microcontroller-based systems expanded by sensory and wireless communication off-the-shelf components). To meet this objective, we discuss technical trade-offs and design decisions, while reporting implementation details relevant to our deployment framework. To our knowledge, the particular structural, architectural and implementation setting adopted in UbiCare has not been reported in the literature. We argue that our experiences may serve as a useful guide for the development of research and commercial AAL or similar tools. It is noted that, besides supporting activity monitoring services, the main design goals of UbiCare also include: low deployment and operational cost; efficient energy management so as to prolong the lifetime of battery-operated nodes; privacy protection through enabling confidentiality across wireless data communications.

The remainder of this article is structured as follows: Section 2 reviews related work. Section 3 presents our experimental testbed and Section 4 discusses functional and technical considerations with respect to our implemented prototype. Lessons learnt from a real-life experiment are documented in Section 5. Finally, Section 6 concludes our work and suggests directions for future work.

## 2. Related Work

During the last few years, a variety of systems have been developed for elderly care, activity monitoring and health-care applications [1]. These systems usually focus on monitoring the activities and the wellness of senior citizens living independently at home or in controlled environments. The types of activities monitored can be categorized in: Activities of Daily Living (ADL), fall and movement detection, location tracking, medication intake and medical status [16]. Rich context information can be obtained by analyzing and fusing various types of sensor data [17]. Depending on their focus, the applications may employ different equipment such as sensor and actuator nodes, cameras, RFID tags, infrared-based Small Motion Detectors (SMDs), MEMS sensors and operation detectors.

Applications for fall and movement detection focus on following user movements and detecting user falls. The application presented in [6] uses an accelerometer located at head-height that can detect a fall and send a notification to a mobile digital assistant. Location tracking applications aim at identifying the location of the user and analyzing his/her behavior. In [18], an indoor positioning system is proposed providing alarm, guidance and leisure services to disabled individuals. The system uses a ZigBee network and an ultrasound-positioning system to perform localization simultaneously by two methods (proximity and multilateration), giving a rough or accurate location as needed.

Medication intake applications focus on monitoring the intake of the patient’s drugs. The iCabiNET solution presented in [19] monitors the intake of prescription and over-the-counter drugs. It employs a smart medicine manager to notify patients via SMS or audio alarms about their medication’s dosages and times. In [10], a healthcare solution for medication noncompliance and ADL monitoring is proposed using an intelligent package sealed by controlled delimitation material with RFID tags; the latter can be detected by an RFID sensor at the moment of ingestion, allowing caregivers to remotely monitor whether or not a patient is adhering to the instructions. Medical status monitoring applications collect clinical data such as heart rate, pulse, glucose monitoring, and elaborate a current-state diagnosis of the patient. In [20], AlarmNet is proposed, an assisted living and residential monitoring network for pervasive adaptive healthcare in assisted living communities with residents or patients with diverse needs. AlarmNet integrates environmental, physiological and activity sensors in a scalable heterogeneous architecture. It monitors a series of variables, stores the data and processes the information to detect any abnormalities.

In [21], a WSN-based application is proposed that focuses on fall/movement detection and on medical status monitoring in a controlled environment to detect falls, tachycardia and bradycardia for the elderly subjects. The application is based on the use of an accelerometer and a heart rate sensor, connected to a mobile monitoring node to collect data and forward it to the WSN infrastructure.

Applications for ADLs focus on monitoring the activities of individuals at home. Elite Care is an assisted living facility equipped with sensors to monitor indicators such as time in bed, bodyweight, and sleep restlessness using various sensors [22]. In [23], an application is proposed which employs RFID cards, a database that keeps a very large number of human activities and a fast inference mechanism that allows the remote identification of a person’s actions. In [9] a system is proposed which utilizes a sophisticated Bayesian network taking input from a series of environmental parameters that can estimate an approximation of the activities. Monitoring activities of an individual using camera-based sensors or fisheye cameras are proposed [7,8] in which the images of the person are taken and analyzed. Systems using accelerometers and RFID communication technology for elderly monitoring in assisted living are reported in [24,25]. Privacy invasive systems certainly require the elderly’s permission; nevertheless, even when used with the consent of monitored individuals, certain social and legal issues may arise and, therefore, are not always acceptable [4].

In [26], a ZigBee-based monitoring system for the elderly is described. Each sensor node is composed of a temperature sensor, a force sensing resistor and a MEMS sensor, an Atmel microcontroller, an LCD module and a ZigBee transceiver. The system logs any changes in the user’s regular daily activities and triggers messages to the care provider about the functional abilities of the user. The monitoring system is noninvasive, low-cost and assesses the elderly activities at home in real-time. In [27], a framework integrating temporal and spatial contextual information for determining the wellness of an elderly has been modeled and behavior detection has been designed. The developed prototype is used to forecast the behavior and wellness of the elderly by monitoring the daily usages of appliances in a smart home.

The systematic examination of existing AAL systems allows identifying several generic trends:

- Recent advances in low-power wireless communication networks have driven a shift towards ZigBee and emerging body area network (BAN) technologies. Technology trends indicate that Bluetooth and RFID will become less common in future AAL prototypes, mainly due to ZigBee’s low energy consumption, enhanced security features and ability to support mesh networks.
- The connectivity of AAL systems with the Internet and the access of remote care providers to activity and medical data via web and/or mobile application interfaces become key design requirements.
- AAL systems increasingly integrate a wide variety of sensors capturing a broad range of contextual parameters. Such sensors tend to be (although are not always) common, affordable devices that can easily be installed in the monitored environment with no significant effort and cost [1].
- Machine vision-based activity recognition becomes less common nowadays in AAL systems, mainly due to the inherent algorithmic complexity of relevant approaches, the proliferation of highly accurate sensor solutions (which easily capture presence and activity data) and the raising of privacy concerns thereby lowering user acceptance [1,28].
- The main focus of AAL research so far has been on system implementation and testing, sensor fusion and activity inference. Tackling challenges beyond technical issues (such as user acceptance problems, usability, user learning and user experience aspects) represents an emerging trend in AAL research [28,29].
- The attention of AAL research community gradually moves from the “traditional” ADL monitoring and fall detection systems to specialized application fields such as: dementia, Alzheimer and comorbidity care; cognitive and kinetic disabilities; heart surgery recovery; hearing impairments; total blindness; and medical emergency detection [30]. In many cases, wearable (body-worn) devices are used, which capitalize on recent advances in MEMS and epidermal electronics for monitoring health parameters (like blood pressure, heart rate, *etc.*) [1].

Table 1 summarizes the main functional and technical characteristics of the AAL systems (listed in chronological order) that are mostly relevant to the scope of UbiCare; the information referring to UbiCare may be found in the last table row.

Evidently, the majority of AAL systems rely on ZigBee-compatible transceivers, which are ideal for battery-operated nodes wherein low cost and long battery life are the key requirements. Moreover, activity monitoring in most existing systems relies on dedicated WSN infrastructures, typically comprised of commercially popular TinyOS-based sensor boards (e.g., MicaZ, iMote2, TelosB motes). Despite the fact that such nodes are commonly based on low-cost components, WSN development kits are comparatively expensive, as are the sensor nodes themselves. The wide variety of sensor nodes available and the limited market size for each product mean that NRE (non-recurring engineering) costs for boards and supporting software dominate the overall system cost [31]. Instead, UbiCare comprises a low-cost prototyping platform based on popular open-source hardware and software environments. In particular, we utilize Arduino microcontrollers coupled with XBee Zigbee networking modules and choose external sensors tailored to the functionality of individual nodes. In addition to cost savings, Arduino offers the following advantages over TinyOS-based nodes: Arduino suggests an extensible ecosystem, wherein the basic circuitry may be expanded by numerous off-the-shelf components (e.g., wireless communication, security, GPS, touch screen and motor control components) to meet specific system requirements; Arduino is ideal for sensing, computation and digital input/output (I/O) tasks, while also providing a well-documented application programming interface (API) which eases the development of complex applications; Arduino microcontrollers far exceed the processing capacity of popular sensor boards, being capable of executing relatively demanding computational tasks; the community support of Arduino allows developers to easily locate coding hacks and facilitates rapid prototyping. On the other side, TelosB, MicaZ, or iMote2 motes prevail with respect to energy expenditure both in processing and transmission tasks [32].

Further to investigating the applicability of microcontrollers as a structural unit for AAL deployments, UbiCare adopts the following design principles, typically not addressed in similar AAL systems:

- It makes no assumptions on the existence of a smartphone or PC/laptop to collect activity reports or emergency data (apart from increasing deployment cost, such requirements would seriously compromise the reliability and availability of the system); rather, this role is undertaken by an inexpensive microcontroller, which only requires an Internet connection.
- UbiCare makes no use of cameras and microphones for activity recognition, acknowledging that such devices are commonly perceived as privacy violators, hence, undermining user acceptance for such systems. Instead, activity monitoring is undertaken by sensor devices which may easily be fabricated within furniture and home appliances, while the monitored subjects are required to carry minimal equipment (waived into their clothes) to mitigate their reluctance in accepting the system.
- Activity detection is based on monitoring the interaction of human subjects with a broad variety of home objects, including furniture, electrical appliances, faucets and sanitary facilities.
- UbiCare addresses the confidentiality requirements of privacy-sensitive activity information taking advantage of the XBee capabilities to enable secure data exchange on both the application and the network layers.
- We have investigated technical solutions to reduce the energy expenditure of XBee modules and battery-operated microcontrollers so as to prolong the system’s lifetime.

**Table 1:** Main functional and technical characteristics of representative ambient assisted living (AAL) systems.

Prototype/Release Date	Application Area	Activity Types Inferred	Sensor Node Platform and OS	Mounted Sensors	Wearable Sensors	Requirement for Additional Computing Infrastructure (to Collect Sensory Data)	Wireless Communication Technology	Support for Remote Monitoring	Unique Functional or Technical Characteristics
Philipose *et al.* [23]/2004	ADL monitoring	Usage of appliances	Not specified	RFID tags on appliances	Gloves and bracelets with RFID tag readers	Not specified	RFID	Yes (activity reports)	Usage of activity models and approximate probabilistic inference to rapidly track those activities.
ITALH [3]/2005	Fall detection; medical emergency detection	Motion events	Telos Rev B Mote (Berkeley)	Temperature, humidity and light sensors	Accelerometer, heart rate monitors	PC, mobile phone	ZigBee, Bluetooth	Yes (display of alarms and incidents of interest, live camera data)	Users explicitly decide who/when has access to health and activity data.
Nasution and Emmanuel [7]/2007	Posture-based events monitoring; fall detection	Standing, sitting, bending/squatting, side laying, laying	-	Camera	-	PC	-	No	Intelligent posture recognition.
Behavior Scope [4]/2008	ADL monitoring	Presence	Intel iMote2	Motion sensors and cameras	-	Server computer	Not specified	Yes (display of notifications)	The system’s architecture offers a flexible, user-configurable time abstraction layer that encodes temporal information in the incoming (sensor) data stream.
Yu-Jin *et al.* [25]/2008	ADL monitoring	Standing, lying, sitting, walking, running	-	RFID tags on everyday objects	Accelerometer, glove with RFID reader (iGrabber)	Smartphone	Bluetooth, RFID	No	Sensor fusion of three accelerometers and a RFID reader.
WASP [11]/2008	ADL monitoring	Usage of appliances & objects, mobility, communication, eating, sleeping, preparing food, leaving & returning home	TinyOS	Microphones, pressure sensors, RFID tags, electricity and water usage sensors, cameras	Accelerometer, pulse oximeter	Mobile phone or PDA, PC	RFID	Yes (display of activity reports & statistics)	Multi-sensor fusion, behavior estimation, interoperation of heterogeneous sensor environments.
AlarmNet [20]/2008	ADL monitoring	Presence in specific rooms, eating, hygiene, sleeping	MicaZ & Telos Sky motes/TinyOS	Temperature, light, dust, motion sensors	ECG, pulse oximeter, accelerometers	Crossbow Stargate SBC	ZigBee	Yes (display of alarms, sensor data, activity reports & statistics)	Extensible heterogeneous network middleware, smart power management, activity pattern learning, dynamic alert-driven privacy.
Zhongna *et al.* [8]/2009	ADL monitoring	Standing, walking *etc.*	-	Cameras	-	Micro-computer	Not specified	No	Efficient silhouette extraction for privacy protection, automated activity analysis.
Putnam *et al.* [5]/2012	Fall detection	-	Arduino	-	Accelerometer	PC	ZigBee	Yes (display of fall events)	Location tracking based on received signal strength.
Ranjitha Pragnya *et al.* [26]/2013	ADL monitoring; wellness monitoring	Usage of household appliances	ARM ATMEL	Temperature, force sensing resistors	MEMS	PC	ZigBee	No	Non-invasive, flexible, low-cost home monitoring.
Suryadevara *et al.* [27]/2013	ADL monitoring; determination of wellness	Sitting, sleeping, usage of household appliances and objects	-	Force sensing resistors, current flow sensors	-	PC	ZigBee	Yes (activity reports & statistics)	Intelligent behavior detection; behavior and wellness forecasting.
WiSPH [21]/2014	Fall detection; medical emergency detection	Motion	WiSe platform [33]	-	Accelerometer, heart rate sensor	PC	ZigBee, Bluetooth	Yes (display of alarms and health status via web interface)	Efficient fall detection algorithm.
UbiCare	ADL monitoring; fall detection	Presence in specific rooms, sitting, sleeping, eating, hygiene, usage of household appliances	Arduino	Motion, force, temperature, humidity, light, electricity and water usage sensors	Accelerometer	-	ZigBee	Yes (display of alarms, activity reports & statistics via web interface)	Unobtrusive and low cost deployment; no requirement for PC, micro-computer to collect sensory data; privacy, wireless security and energy saving considerations are addressed.

## 3. Experimental Testbed

**Figure 1:**
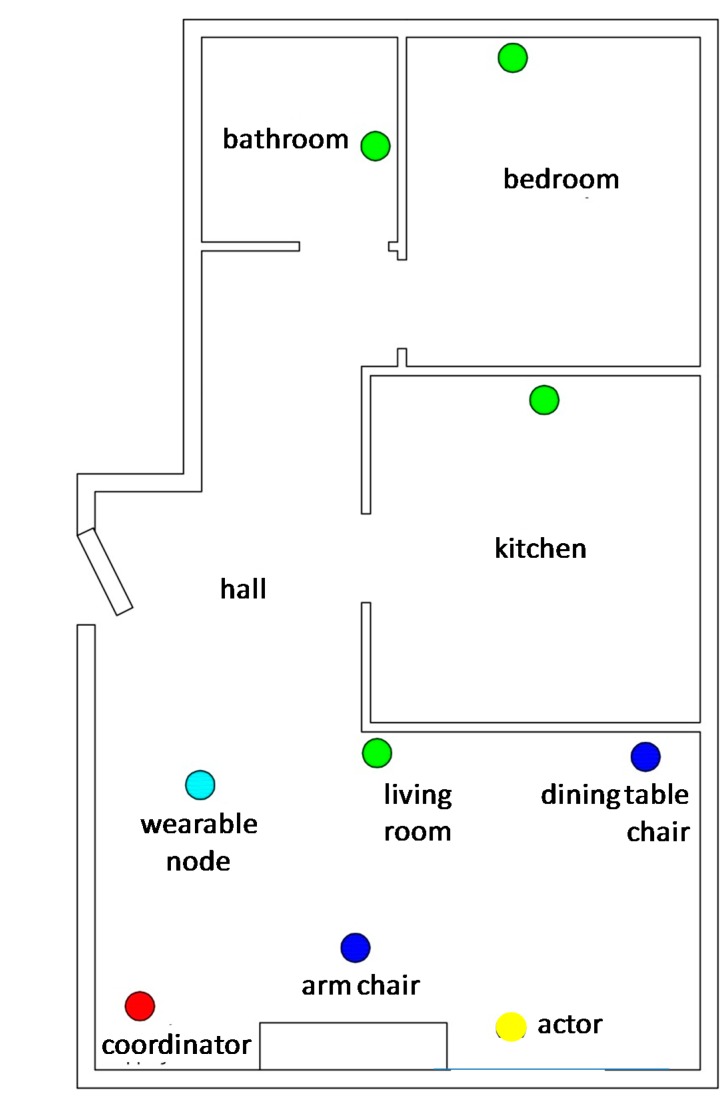
Plan view of the UbiCare deployment environment.

Our prototype system has been deployed in a controlled part of a house and comprises a WSN of nine nodes: four nodes installed in the main rooms of the house (bedroom, bathroom, kitchen, living room), two nodes mounted on furniture (armchair and dining chair), a wearable node, an actuator and a coordinator node. A plan view of the controlled home environment is illustrated in Figure 1.

**Figure 2:**
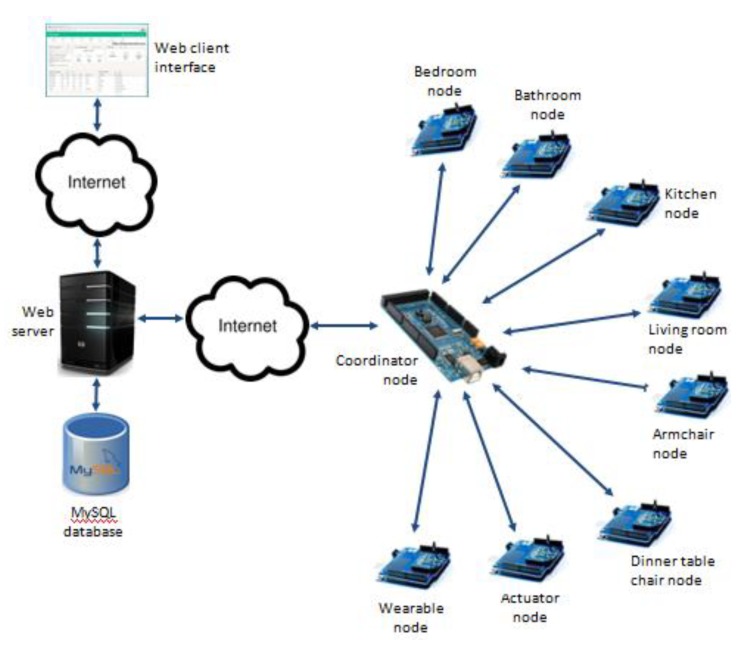
Experimental testbed architecture of UbiCare.

Out of the above mentioned nodes, the ones installed in the rooms and the furniture are used to collect environmental parameter values (e.g., temperature and light intensity) and activity information (e.g., the elderly entered the kitchen, opened the fridge, laid on the bed, flushed the toilet or sat on the chair). The wearable node is worn on the elderly’s arm and is programmed so as to detect falls, also allowing calling for help in emergency situations (through pressing a “panic” button). The actuator node is used to remotely (either in manual or automated fashion) control any electrical device (e.g., automatic operation of the fan depending on the room temperature). The coordinator node is enrolled in the collection/processing of activity information as well as uploading the information to a web database, making it accessible via standard web interfaces. Figure 2 illustrates the UbiCare testbed architecture.

Table 2 elaborates on the types of captured activity status as well as on the hardware modules utilized to build the sensor nodes and their respective (approximate) costs.

**Table 2:** Types of activity status monitored and hardware integrated on sensor nodes of UbiCare along with their respective cost.

Node	Activity Type	Hardware	Cost
Bedroom node	Movement Presence on bed Step upon the mat next to bed Light intensity Temperature, humidity Panic status	Arduino UNO XBee RF Module (+XBee shield) Prototype shield Motion detector Force sensing resistor Light intensity sensor Temperature and humidity sensors Panic status button Electronic components and breadboard Power adapter	$97
Living room node	Movement Light intensity Temperature, humidity	Arduino UNO XBee RF Module (+XBee shield) Prototype shield Motion detector Light intensity sensor Temperature and humidity sensors Electronic components and breadboard Power adapter	$67
Kitchen node	Movement Use of electric appliances (microwave oven, oven, toaster, kettle, fridge), drugs cabinet and sink’s faucet Light intensity	Arduino UNO XBee RF Module (+XBee shield) Motion detector AC/DC current sensor Light intensity sensor Water flow sensor Magnetic contact (reed) switch Electronic components and breadboard Power adapter Electrical equipment and plumbing components	$73
Bathroom node	Movement Use washbasin’s faucet and toilet’s flasher Light intensity Panic status	Arduino UNO XBee RF Module (+XBee shield) Prototype shield Motion detector Light intensity sensor Water flow sensor Panic status button Electronic components and breadboard Power adapter Plumbing components	$65
Arm chair node	Presence (sitting) Panic status	XBee RF Module (+XBee breakout board) Force sensing resistor Panic status button Breadboard Batteries	$35
Dining table chair node	Presence (sitting)	XBee RF Module (+XBee breakout board) Force sensing resistor Breadboard Batteries	$34
Wearable node	Panic status Fall	Arduino Lilypad XBee module (+XBee breakout board) Accelerometer Panic status button Battery	$83
Actuator node	-	XBee RF Module (+XBee breakout board) Relay Electronic components and breadboard Batteries Electrical equipment	$29
Coordinator node	-	Arduino Mega XBee RF Module (+XBee shield) Ethernet shield Power adapter	$98

The total acquisition cost for the hardware required to implement the prototype nodes has been ~$581 (Fall 2014).

## 4. Implementation Issues

### 4.1. Hardware

UbiCare network nodes have been implemented by gluing together independent modules: microcontroller boards, wireless communication modules, various kinds of sensors, power supply, *etc*. Arduino (Arduino systems provide sets of digital and analog I/O pins that can be interfaced to various extension boards and other circuits. The boards feature serial communications interfaces, including USB on some models, for loading programs from personal computers. For programming the microcontrollers, the Arduino platform provides an integrated development environment (IDE). Software is authored in the Arduino programming language, which is based on the Processing multimedia programming environment project.) has been chosen as a microcontroller platform as it comprises an open-source, flexible, low-cost platform (Nowadays, the rapidly evolving market of microcontrollers and microcomputers features several alternatives to Arduino (many of them Arduino clones) for deploying AAL systems, such as BeagleBone [34], Raspberry Pi [35], Nanode [36] and Libelium Waspmote [37]. However, Arduino is advantageous over competitive boards with respect to: significantly lower cost; large community support and availability of large pool of libraries which facilitates software development; higher flexibility and extensibility due to the hardware openness and the availability of various shields; availability of several Arduino board variants, each of which may address specific application needs in terms of size, memory resources, power consumption, number of input/output pins, *etc.*). To provide an indicative measure of comparison, note that the acquisition cost of Libelium Waspmote nodes, with equivalent specifications to the UbiCare bedroom, living room and kitchen nodes (as specified in Table 2), would be $243, $213 and $219, respectively, whereas the total acquisition cost would be $1400 (*i.e.*, 140% higher than the Arduino-based solution) (We have chosen to compare the cost of our Arduino-based deployment against the Waspmote platform, as the latter represents the most complete out-of-the-box solution, purposely designed to support relevant applications, also addressing energy consumption and wireless communication security issues; hence, a Waspmote-based solution would be directly comparable to the deployment proposed in this article. Note that, the utilization of Waspmote extension modules which include groups of sensors (rather than plain low-cost sensors) would further increase the cost of Waspmote nodes.). Furthermore, Arduino considerably simplifies the amount of hardware and software development needed to setup sensing and control applications. The Arduino hardware platform comes with circuitry to program and communicate with the microcontroller. On the software side, Arduino provides a number of libraries to facilitate the microcontroller programming, e.g., to control and read the I/O pins, set I/O pins to PWM (Pulse Width Modulation) values at a certain duty cycle using a single command or via serial communication. Arduino and Arduino-compatible boards use shields—printed circuit expansion boards plugged on top of the Arduino PCB (printed circuit board) extending its capabilities (e.g., I/O expansion shield, Ethernet shield, relays shield, GPS shield, Bluetooth shield, SD card shield, *etc.*). It is noted that the majority of UbiCare nodes utilize Arduino microcontrollers powered through a power adapter. The exceptions are the wearable, the actuator, the arm chair and the dining table chair nodes, which are battery-operated. This implementation decision has been taken because the actuator does not require any data processing, while the wearable as well as the chair nodes have strict portability requirements which prohibit the use of power cords (The prototyping of sensor nodes using a microcontroller enables the processing of sensory data and the uploading of information to the sink only upon detecting specific events (the lack of microcontroller necessitates the frequent, periodic uploading of row data, thereby increasing the use of bandwidth resources and the energy consumed for data transmission). On the other hand, the use of the microcontroller increases the purchase cost, increases the nodes’ size and weight (hence, limits its portability) and poses higher energy requirements, dictating the use of a power adapter.).

**Figure 3:**
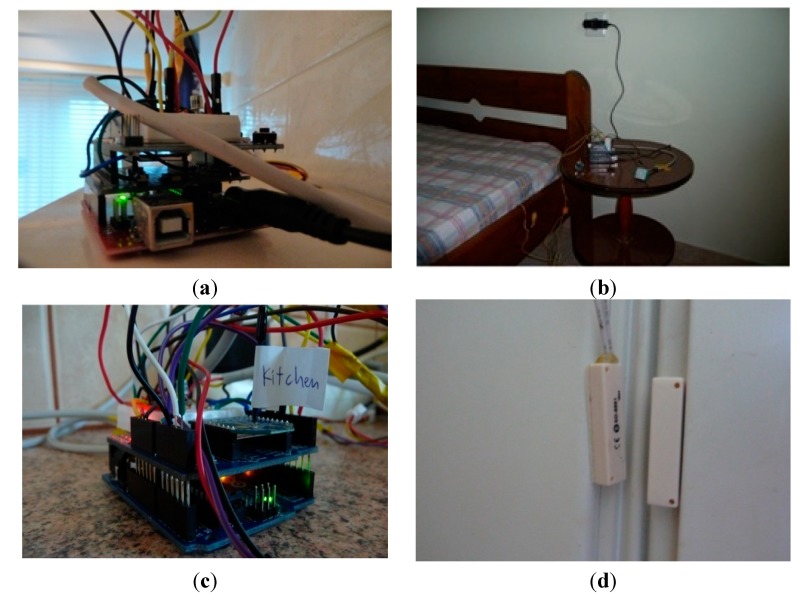

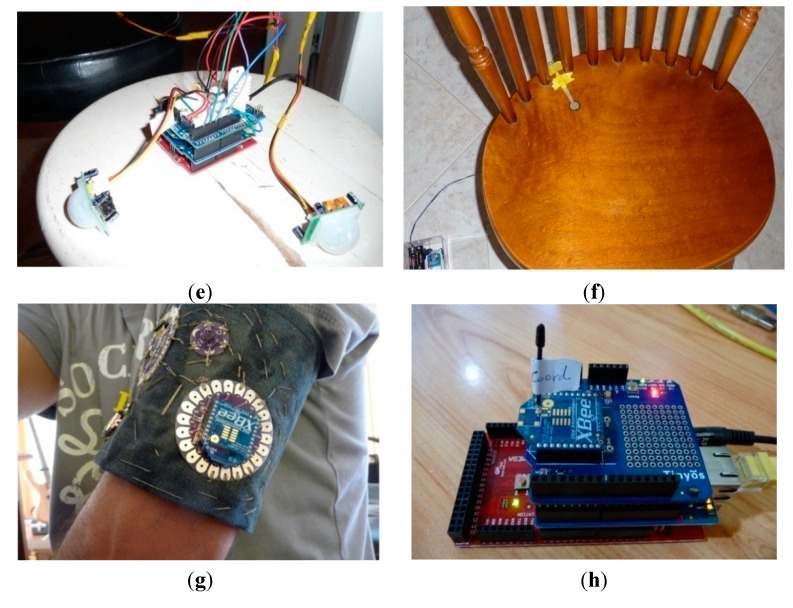
(**a**) The bathroom node; (**b**) the bedroom node; (**c**) the kitchen node; (**d**) magnetic contact switch mounted on the fridge; (**e**) the living room node; (**f**) the dining table chair’s force sensing resistor and node; (**g**) the wearable node; (**h**) the coordinator node.

The wireless communication among the nodes and the processing element is undertaken by low-range, ZigBee-compatible XBee RF modules. ZigBee modules are available at lower cost, while also reducing power consumption (therefore, energy requirements) compared to alternative wireless technologies. Moreover, ZigBee supports mesh networks, low duty cycle, low latency communication and 128-bit security. Figure 3 shows pictures of representative UbiCare nodes and sensors.

**Figure 4:**
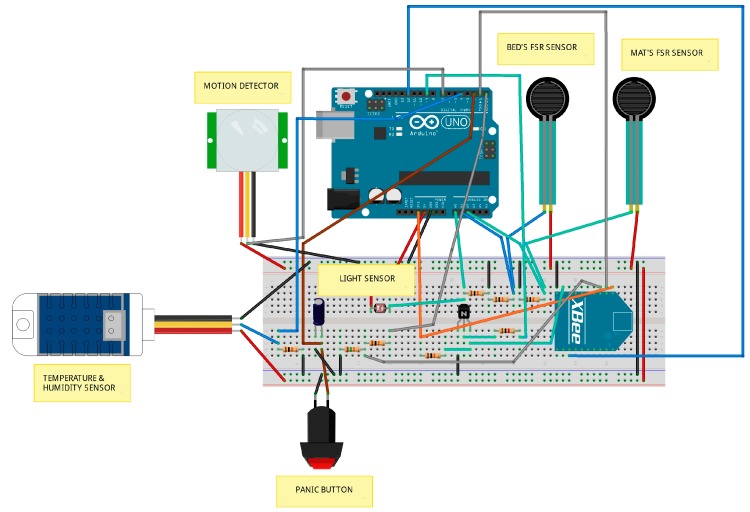
Illustration of the hardware components and wiring of the bedroom node.

**Figure 5:**
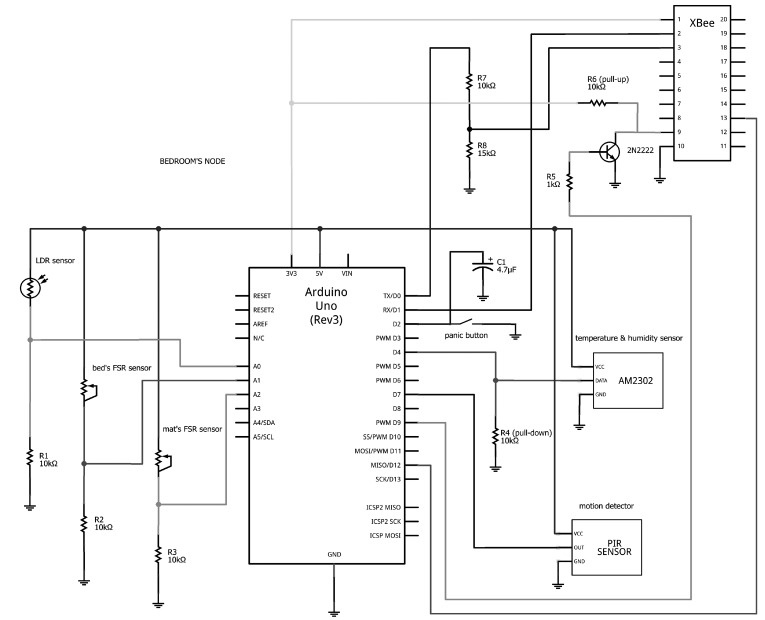
Schematic representation of the bedroom node’s pin connections.

Figure 4 illustrates the hardware components and wiring of a representative UbiCare node. The corresponding schematic representation of the node’s pin connections is presented in Figure 5.

### 4.2. Software

We have developed software (based on the Arduino programming language [38]) to program network nodes, tailored to the role and functionality of each node. In particular, the software executed by microcontrollers undertakes the tasks of sensory data collection/processing and information transmission to the wireless medium (either when the measured parameter values change or a specific period of time elapses). The sensory information is received and processed by the coordinator node which infers activity incidents; thereafter, incident reports are forwarded to the web server. It is noted that AAL tools are commonly supported by sophisticated algorithms and computational techniques to enable accurate activity recognition, planning and anomaly detection. As our main focus has been on the actual system implementation and deployment, UbiCare currently only supports simple rule-based activity inference; namely when specific conditions are known, then certain conclusions are inferred. For instance, when the toaster is switched on/off early in the morning and then a subject sits on the dinner table chair, it is inferred that a resident is having breakfast.

**Figure 6:**
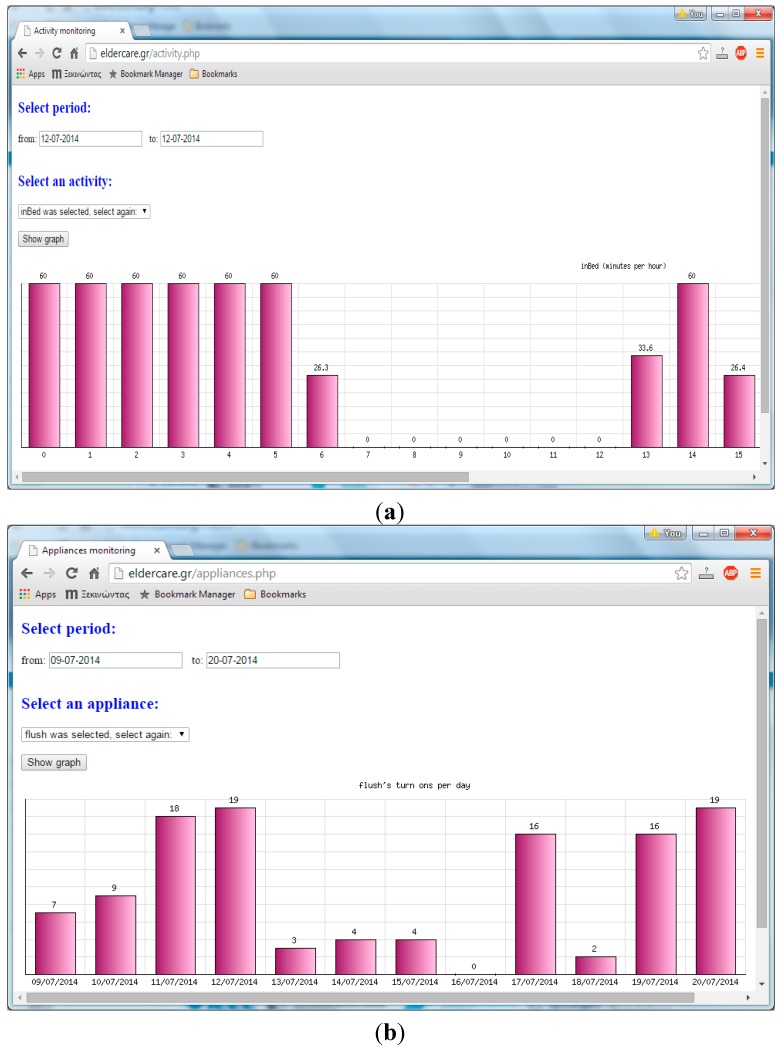
Visualization of activity monitoring; (**a**) Time spent (minutes per hour for a selected day) on bed; (**b**) toilet flush activation occurrences per day.

Furthermore, we have developed web (PHP) software that allows remote monitoring of elderly residents via a standard web interface [39] (The database maintains activity statistics for the period 9 July to 26 October 2014.). This software undertakes the storage and management of sensory data in a MySQL database, exports statistics and creates dynamic graphs. Authorized end-users (typically, caregivers) are allowed to view current and historical data about: temperature, humidity and light level values; activity (presence, laying on bed, standing next to bed, sitting on chairs); number of activations for electric appliances (oven, microwave oven, toaster, kettle, fridge); usage of faucets, the drugs cabinet, the flush and the actuator; batteries voltage level (only for battery-operated nodes); triggered alarms (due to fall/panic incidents and node failures). The user may also remotely control the actuator node, for instance switch on/off an electric fan. Figure 6 illustrates examples of activity monitoring visualization.

### 4.3. Energy Management Issues

The lifetime extension of battery-operated nodes is a crucial aspect of AAL systems, as seniors cannot be reasonably expected to frequently check the residual battery life or replace batteries [40]. Along this line, the following two subsections present practical guidelines in addressing energy management issues on the microcontroller platforms and the energy-hungry communication (XBee) modules, respectively, to allow deploying extremely energy-efficient systems with dependable battery life.

#### 4.3.1. Energy Management in Microcontroller Platforms

Most Arduino boards (e.g., Uno, Mega) are inappropriate for battery operation since they integrate energy consuming hardware which soon depletes the reserves of commercial batteries. For this reason, all the UbiCare prototype nodes which include an Arduino board (except of the wearable node) use a power adapter.

Significant battery power savings can be achieved by setting the Arduino into sleep mode either for a predetermined period of time or until an input pin changes through external interrupts. In our prototype, sleep mode is exclusively implemented in the Arduino LilyPad of the wearable node (using the Narcoleptic library [41]), since this is the only battery-operated microcontroller. An input pin of the LilyPad is connected with a panic button to enable awaking of the node by the user in emergency situations. The sleeping period has been set to 100 ms so as to ensure that no fall will be missed. Our measurements have shown that the use of sleep mode drastically decreases energy consumption (from 3.5 mΑ down to a few μA).

#### 4.3.2. Energy Management in ZigBee (XBee) Modules

XBee modules support a number of sleep modes (SM) which enable the RF module to enter states of low-power consumption (when not in use) and achieve considerable energy savings. Therefore, the use of sleep modes is crucial for energy conservation in battery-operated nodes. By default, sleep modes are disabled (SM = 0), and the module remains in idle/receive mode. When in this state, the module is constantly ready to respond to serial or RF activity. The sleep modes most commonly used in XBee modules are the following:

- Pin Hibernate (SM = 1), which minimizes the quiescent power (power consumed when in a state of rest or inactivity) at the expense of longer wake-up time (~13 ms). This mode is voltage level-activated; to wake up a sleeping module operating in Pin Hibernate mode, pin #9 should be de-asserted. This mode is appropriate to use when sleep transition is controlled by an external microcontroller (such as Arduino).
- Cyclic Sleep Remote (SM = 4), which allows modules to periodically check for RF data. In this mode, the module is configured to sleep and send a poll request to the coordinator at a specific interval set by the SP (Cyclic Sleep Period) parameter. The coordinator then transmits any queued data addressed to that specific module. The module returns to sleep mode in the case that it detects no radio activity for a period “Time Before Sleep” (ST). It is noted that this mode is more energy consuming for sleeping modules compared to the hibernate mode; however, the wakeup time is only 2 ms.
- Cyclic Sleep Mode with Pin Wake-up (SM = 5), which allows the wake up of a sleeping module either periodically or by the de-assertion of pin #9 for event-driven communications.

Table 3 presents the sleeping mode configurations for our prototype XBee modules. Evidently, the energy-efficient pin hibernate mode has been used in all nodes where the RF module is controlled by an Arduino. The armchair node’s RF module has been set to “Cyclic Sleep/Pin Wakeup” mode so as to periodically check the armchair’s status (*i.e.*, whether someone sits on it) and also allow immediate transmission in the case that the panic button (connected to the XBee module’s pin #9) is pressed by the user. In the remainder nodes (dining table chair, actuator), we have used the cyclic sleep mode with the settings representing a compromise among energy conservation and immediacy of event detection (for instance, if someone sits on the dining table chair for a period less than the sleep period (Note that the armchair and the dining table chair nodes have been set to extended cyclic sleep mode. Therefore, the sleeping period for their XBee modules is 3.2 × 20 = 64 s. This is a reasonable choice since the elderly individuals are expected to stay seated for much longer periods. In contrast, the actuator node’s XBee module has been set to short cyclic sleep mode, therefore, its sleeping period is 10 s.), the sensor node is likely to miss this event).

**Table 3:** XBee modules sleep mode settings.

Node	Sleep Mode	Time before Sleep	Cyclic Sleep Period	Number of Cycles to Power down IO	Sleep Options
coordinator			SP = AF0 (28 s)	SN = FFFF (65.535)	
bedroom	SM = 1				
bathroom	SM = 1				
kitchen	SM = 1				
living room	SM = 1				
armchair	SM = 5	ST = 3E8 (1 s)	SP = 140 (3.2 s)	SN = 14 (20)	SO = 0 × 04 (extended sleep)
dining table chair	SM = 4	ST = 3E8 (1 s)	SP = 140 (3.2 s)	SN = 14 (20)	SO = 0 × 04 (extended sleep)
actuator	SM = 4	ST = 3E8 (1 s)	SP = 3E8 (10 s)		
wearable	SM = 1				

### 4.4. Privacy Issues

Privacy and confidentiality represent other major issues that should be seriously considered in the design specifications of AAL devices [42]. All communications should be encrypted and secure to ensure confidentiality of the sensitive activity data. This is especially important in the case of wireless communications which are easier to intercept.

Fortunately, the ZigBee specification supports message confidentiality and integrity on both the network and application layers, thereby addressing the basic privacy requirements of AAL systems. Network layer security protects wireless communications on each packet forwarding, while application layer security is implemented on the communication ends to protect application data [43,44]:

- On the network layer ZigBee implements: 128-bit AES (Advanced Encryption Standard) symmetric key encryption on packets payload, using a network key (NK); data integrity check through applying a hash function on packets’ header and data, using the NK; protection against replay attacks through using a 32-bits frame counter.
- On the application layer, ZigBee implements secure communication for the application data exchanged among two end devices, through applying an encryption key (link key, LK).

It is noted that the NK is common among all network devices (The NK is generated by the Trust Center (typically, the coordinator node) and regenerated at different intervals), whereas LKs are unique for each pair of nodes (LKs are securely exchanged between two nodes through the Key Establishment Procedure (SKKE). SKKE requires “master keys”, which are pre-installed in each node (their function is to ensure confidentiality in LK exchange).). In UbiCare, we have taken full advantage of the security mechanisms of ZigBee specification on the network and application layer [45]:

- The network key of the coordinator node has been set to NK = 0, so that a random network key will be selected. A ZigBee device can join the network subject to obtaining NK from the coordinator.
- The link key has been set to the same (arbitrary) value (ΚΥ > 0) on all devices (the coordinator and the end devices). Upon successfully joining the secure network, all application data transmissions will be encrypted by the (randomly chosen) network key. Since KY > 0, the network key is sent in encrypted form by the pre-configured link key (KY) when the devices join the network (When setting KY = 0 on all devices, the NK is sent unencrypted (“in the clear”) to joining devices. This approach introduces security vulnerability into the network and is not recommended.).

## 5. Real-Life Experiment and Lessons Learnt

We have tested and conducted a real-life experiment with UbiCare in the period of July–October 2014. The subjects (residents) were a couple in their seventies. They were not researchers and their involvement in our research has been voluntary. UbiCare has been deployed in their summer-house. The researchers’ team monitored the system’s operation online through the web interface. Samples of the online reports have been validated via telephone communication with the subjects.

UbiCare operation has been smooth with regards to the system’s availability. To a sufficient degree, activity reports have been accurate throughout the reported period. Nevertheless, the following malfunctions have been identified during the testing and trial period:

- Occasionally, the wearable node incorrectly reported sudden movements as falls. Likewise, the node failed to detect slow falls on several tests. Addressing such inaccuracies is not straightforward [5] and requires: (a) thorough investigation as regards the optimal body position to place the node (e.g., hip or waist) [46]; (b) testing of different fall detection algorithms on several real ADL datasets; (c) mounting of additional sensors on the wearable node, such as gyroscope and altimeter.
- Moving events from one room to another have not been accurately detected in all occasions. In all probability, such inaccuracies could be addressed by using a larger number of motion detectors per room (e.g., covering different angles). Likewise, the force sensing resistor (FSR) sensors occasionally missed some events (lying on bed and stepping upon the mat next to bed), mainly when a subject lied on the edge of the bed or pressed the edge of the mat. Such problems could be easily tackled by using more than one FSR sensor to detect pressure activity.
- On rare occasions, events wherein the subject sat on the dining table chair or the arm chair and then shortly got up could be missed (provided that the events took place while the corresponding nodes were in sleep mode). However, this is not regarded as a problem, since this kind of events would be captured by decreasing the time the respective nodes remain in sleep mode, at the expense of increasing battery consumption.

Moreover, the real-life experiment highlighted a number of practical considerations:

- The choice of a suitable quantity and type of sensor for monitoring a certain activity is not straightforward (for instance, we have found that water flow sensors are not adequately reliable for detecting “turning on” events for taps), but requires careful investigation and thorough testing. This choice is dictated by a number of factors, such as cost, reliability, accuracy and installation effort.
- The necessity of using a microcontroller platform on a sensor board should be examined per case and could be determined by several factors, like: the need to perform some kind of information processing; the complexity/time associated with software development; the available budget for the node and the entire system; requirements related with the size, weight, energy efficiency and portability of the node.
- The considerable size of the Arduino-based nodes compromises the unobtrusiveness and discreteness of the overall deployment and demands extra effort and creativity to hide the boards and the accompanying wiring.
- To enable sophisticated activity inference on the spot and immediate triggering of actions on certain actuators, a relatively powerful host would be required (connected with the coordinator node via a LAN) as the Arduino MEGA would not have the capacity to support computationally challenging tasks.

We have edited various video demonstrations illustrating: the experimental setup of our prototype [47]; the online presence and activity monitoring [48]; the triggering of a fall alarm [49]; the remote controlling of the actuator node via a web browser [50].

## 6. Conclusions & Future Research

We presented UbiCare, an AAL system supporting activity detection, fall detection and well-being promotion for elderly individuals. We have addressed practical implementation aspects for deploying low-cost AAL systems comprising microcontroller-based nodes expanded by communication, sensory and actuation off-the-shelf components. We documented experiences and “how-to” design and implementation decisions which may be a useful guide for practitioners and researchers of the AAL community. Particular emphasis has been given in technical solutions addressing two critical aspects of AAL tools: wireless communication security and energy management for battery-operated sensor nodes.

Our future research plans include the following aspects:

- Implement prototype extensions to cover other aspects of well-being surveillance, such as air quality monitoring to detect fire or high concentration of carbon monoxide or automated control of air quality/cleaning devices. Further, actuator nodes could be used to display reminders in the event of anomaly detection (e.g., to switch off the oven or to take medication).
- Integrate a computational infrastructure to enable deriving more abstract (*i.e.*, higher level) information, which may be processed by context-aware application components [51,52].
- The deployment of real AAL systems engages substantial labor and financial investments; this highlights the increasing importance of simulations in AAL research [53,54]. Along this line, the use of AAL-tailored simulators would enable thorough experimentation with the architectural and technical elements of UbiCare under different application scenarios and environmental configurations.
